# Research on the Assembly of Square Parts on Circular Production Lines Using Admittance Control for Six-DOF Robots

**DOI:** 10.3390/s25041138

**Published:** 2025-02-13

**Authors:** Zhiyuan Chai, Junhua Chen, Hao Li, Wenping Xiang, Dongdong Chang, Zewen Liu

**Affiliations:** 1 College of Science and Technology, Ningbo University, Ningbo 315300, China; chaizhiyuan@stu.wzu.edu.cn (Z.C.); lihao1@nbu.edu.cn (H.L.); 2211170028@nbu.edu.cn (W.X.); 2211170017@nbu.edu.cn (D.C.); 2211170024@nbu.edu.cn (Z.L.); 2 Faculty of Mechanical Engineering and Mechanics, Ningbo University, Ningbo 315211, China

**Keywords:** compliant assembly, vertical hole searching, complex parts, impedance parameters, admittance control, circular production lines

## Abstract

In circular production lines, issues such as positional errors, manufacturing errors at different square groove workstations, and the accumulation of errors during continuous assembly reduce the assembly success rate and limit efficiency. To address these challenges in assembling square groove parts, this study focuses on the jamming and wedging problems encountered during the assembly of square grooves and square parts. It analyzes typical assembly hole searching contact situations and proposes corresponding strategies. Based on the relative geometric relationships between the assembly workpieces, the entire assembly process is divided into three stages: pre-assembly, hole search contact adjustment, and insertion. Due to the complexity of predicting assembly forces and the uncertainty of workstation assembly in circular production lines, this study emphasizes the hole search contact adjustment stage. An innovative vertical hole search strategy is proposed and compared with the Archimedes spiral search method. This strategy models the contact between the end effector and the environment as a mass–damping–spring second-order system, achieving compliant assembly of square holes through admittance control. By analyzing the admittance parameters using control variables, the optimal admittance parameters are determined, and the admittance parameter pattern is applied in the experiments. Experimental results on the square hole assembly platform show that, under the optimal admittance parameters, the vertical hole search strategy significantly reduces search time and improves efficiency. Compared to the traditional Archimedes spiral hole search strategy, the average search adjustment time was increased by 5.8 s, improving efficiency by 46.4%, and the desired assembly outcomes were achieved.

## 1. Introduction

Since the 1960s, industrial robots have increasingly been applied in various fields across industrialized countries. In manufacturing, applications span areas such as raw material processing (stamping, die-casting, forging, etc.), machining, welding, heat treatment, surface coating, polishing, material handling, assembly, inspection, and warehouse stacking, significantly enhancing processing efficiency and product consistency. Position control of industrial robots can address tasks such as machining, loading and unloading, and other operations. While simple engineering problems can be solved with standalone visual sensors, using visual sensors for hole detection has the drawback of not providing sufficient positioning accuracy. This is because visual sensors need to be combined with force sensors, and there are challenges such as complex programming and the high cost of visual systems, which limit their use. Therefore, in fields such as surface coating, grinding, polishing, assembly, and ultrasound scanning in medical robots [1], relying solely on position control or visual sensors is inadequate for performing tasks with precision. Force control is needed to address the issue of contact force during the operation. With the intensification of population aging and a significant increase in labor costs, the demand for robotic compliant assembly is growing. Therefore, the study of compliant control in industrial robots has considerable research prospects and practical significance today [2].

Compliant control can be divided into active [3] and passive compliance [4] and composite compliant control [5]. The earliest passive compliance mechanism was proposed by Whitney et al. [6,7] in 1986, known as the Remote Compliance Center (RCC) device. This device consists of multiple springs, and by controlling the stiffness of the springs as a variable, it allows the mechanism to achieve different compliance properties. Subsequently, improvements led to the development of SRCC [8], VRCC [9], DRCC [10], and others. Some foreign scholars, such as Chen et al. [11] have introduced a planar flexible parallelogram linkage mechanism, which is an effective method for accurately predicting the static motion of robotic arms. In China, researchers like Deng Xiaoxing [12] and Yuan Shui [13] have proposed passive compliant grippers and tolerance-compliant hands, which can generate minimal contact forces between shaft holes during assembly and compensate for larger deviations between assembly parts. However, these are only suitable for small load applications at the end effector and lack universality. In active compliance control, it can be categorized into impedance control and force–position hybrid control [14]. Force/position control encompasses various approaches, including hybrid control [15], adaptive control [16], robust control [17], neural network control [18], and learning-based F/P control [19,20,21]. Han M et al. [22] developed a force–position hybrid control [23] for parallel mechanisms and proposed a hybrid synchronization control method based on cross-coupling. Feedback information is typically obtained through six-dimensional force/torque sensors to achieve compliant control during assembly operations. Some domestic researchers have adopted sensorless methods to complete assembly tasks and have made progress. For example, Park from Seoul National University [24] proposed an inexpensive peg-in-hole assembly method based on environmental geometric constraints, completing the assembly task without force sensing or with minimal sensing feedback. The disadvantage of these sensorless or low-sensor methods is their generally poor precision, which makes them unsuitable for high-precision assembly tasks. Other scholars have used intelligent algorithms to train models and support experimental research with comprehensive datasets. For instance, Roveda L [25] and others have established reinforcement learning-based assembly models [26,27,28,29], although these approaches require the collection of substantial environmental contact information. Ahn et al. [30] proposed a novel assembly strategy based on visual and force information. The strategy trains trajectory generators based on visual and force data using both simulation data and manual teaching, and the optimal assembly strategy is obtained through deep reinforcement learning algorithms. However, this method relies on good background image segmentation, making it suitable only for assembly scenarios with simple backgrounds. Professors Xu Guangyou et al. [31] from Tsinghua University and Song et al. [32] have also improved assembly success rates by extracting complex part data from CAD models, but these methods lack generality and are difficult to promote. Additionally, there are also active compliance control methods designed for constant force polishing scenarios [33,34]. Li et al. [35] proposed an optimal sliding mode control method for trajectory tracking of discrete-time systems based on a linear quadratic regulator, and used MATLAB/Simulink to simulate the state trajectories of upper-limb rehabilitation robots under various weighting values. In the field of assembly, Norberto et al. [36] demonstrated that it is feasible to generate the kinematic and dynamic models of a robotic manipulator starting from CAD design, create the robot, and develop Simulink modules for the simulation platform.

**Figure 1:**
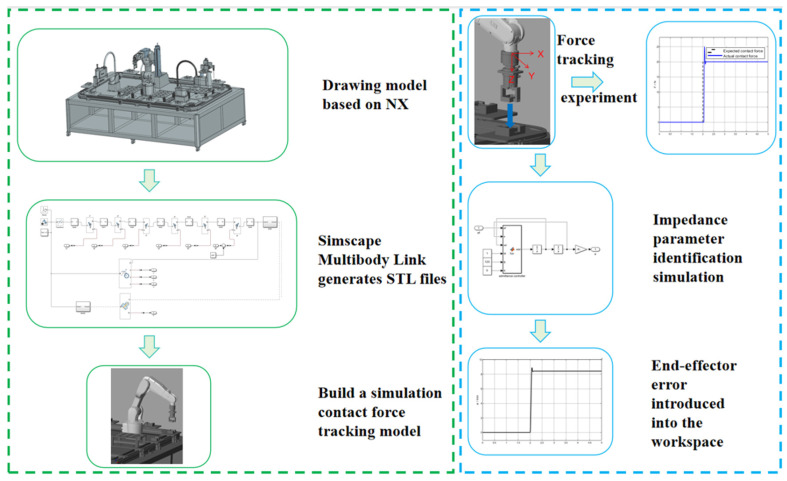
Virtual simulation platform.

This paper investigates hole-based compliant assembly using position-based impedance control, analyzing the impact of impedance parameters on the impedance model control. The feasibility of the proposed method is further validated through assembly experiments. To meet the assembly task requirements, a combined simulation platform is established using Simulink and NX 2206 to construct rigid body 3D models and perform robot admittance control simulations. The developed simulation control system mainly consists of three parts: creating the virtual environment, reconstructing the robot 3D model, and recording simulation data. Different modules are built according to specific needs to achieve modular encapsulation. As shown in Figure 1, the first step is to establish the production line model for assembly, which includes the ABB IRB1200 robot body, 12 sets of square slot stations, square sealing boxes, six-dimensional force sensors, and two connecting flanges that link the six-dimensional force sensors to the robot’s end effector and gripper. Next, the robot model is built in MATLAB R2024a, where a link coordinate system is established. The links and joints are connected according to the coordinate transformations between the robot joints, and the 3D model is imported into the Solid block with its parameters set. For the subsequent compliant control of the square slots and square sealing parts, impedance parameter analysis is performed. The optimal parameters are obtained using the control variable method, which will be applied to the actual assembly experiment. Additionally, a simulation of the force tracking experiment along the *Z*-axis was conducted, which yielded good response speed and force tracking performance, thus laying the foundation for future experiments.

## 2. Complex Part Assembly Situation

When it comes to the assembly of complex parts, the complexity arises from the intricate and specialized geometry of their outer contours, which makes the assembly process extremely complicated. The contact forms during assembly are also more diverse. The assembly process requires adjustments in both directions and orientation in order to achieve compliant assembly. To study the assembly issues of complex parts, we first take the example of a square hole groove and square parts for research, thereby achieving a “teaching by analogy” effect.

### Typical Failure Analysis of Square Hole Assembly

Research on the assembly of square seal boxes and square slots in intelligent assembly production lines is virtually nonexistent. Most international research has focused on the assembly of round hole pins, with only a few scholars exploring square holes and square parts. For instance, Park et al. [37] proposed a human behavior-inspired intuitive assembly strategy; however, its applicability is limited to laboratory settings, and it has not been experimentally validated. Kim et al. [38] studied assembly using shape recognition algorithms. However, this method has the disadvantage of being cumbersome, requiring the collection of complex datasets, and being susceptible to distortion due to environmental changes.

**Figure 2:**
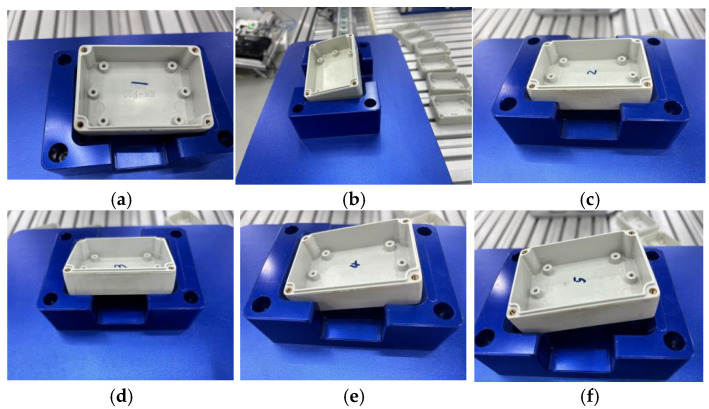
Typical failure modes of square hole assembly. (**a**) Adjacent two-point contact situation. (**b**) Three-point contact situation with one bottom and two sides. (**c**) Four-point interference contact situation on the side surface. (**d**) Line contact situation on the side surface. (**e**) Two-point contact situation with one bottom and one side. (**f**) Two-point contact situation on the chamfered inner side.

The assembly of square holes is inherently more complex than that of round holes due to the special geometry of the parts’ outer contours, leading to more intricate contact forms during the assembly process. Depending on the number of contact points, various types of contact may occur, including single-point contact, multi-point contact, or even line contact. These different contact forms can complicate the identification of force signals, thereby increasing the difficulty of the assembly process. Figure 2 below lists some of the typical assembly failure scenarios encountered.

## 3. Hole Search Strategies Analysis

### 3.1. Archimedes Spiral Hole Search

**Figure 3:**
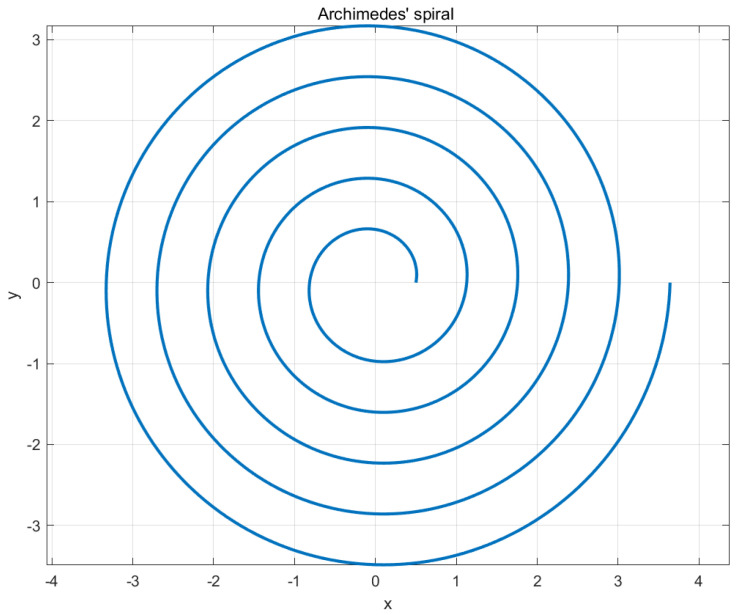
Archimedes’ spiral diagram.

Typical hole search strategies are generally classified into spiral hole search, grid hole search, and random hole search. The most common approach for spiral hole search is the Archimedes spiral search, with the spiral path diagram shown in Figure 3. Its polar coordinates can be expressed as follows:

(1) r=a+bθ

where a is the initial radius of the spiral, b is the spacing between adjacent spiral lines, and θ is the angular increment of the spiral path. In practical hole search experiments, the trajectory is often planned using Cartesian coordinates, and the equation for the path is as follows:

(2) $$\left.\begin{array}{c} x=x_{0}+bcos\alpha \\ y=y_{0}+bsin\alpha \end{array}\right\}$$

where $x_{0}$ and $y_{0}$ represent the starting position of the hole search. In the equation, (x,y) represents the target point to which the robot needs to move. Since the *Z*-axis coordinate is constant, it is not shown in the trajectory equation. Both θ and b are expressions that include T, where T ranges from 0 to 500 with an interval of 1, meaning the trajectory equation contains 500 points. The initial value is set to 60°. Due to the small clearance between the shaft and the hole components, the maximum search diameter is set to 1 mm.

To validate the improved hole search strategy proposed in this paper, a comparison is made with the Archimedes spiral hole search strategy under the same experimental conditions, specifically comparing the time required for the assembly of a sealed box. The following Figure 3 below shows the hole searching trajectory of the Archimedean spiral, which is plotted in MATLAB based on the theoretical equation mentioned above.

### 3.2. Working Principle of the Vertical Hole Search Strategy

The vertical hole search strategy is a method in which the robot’s end effector position is adjusted in the vertical direction to locate and align with a hole. During the assembly process, the robot needs to precisely align the hole on the square box with the target position. To ensure efficient and accurate mating, the vertical hole search strategy adjusts the robot’s end effector posture based on external force feedback, allowing it to locate the target hole and perform the mating.

The working principle of this strategy typically includes the following steps:

(1) Force Feedback Perception:

During the assembly process, the robot continuously monitors the contact force between its end effector and the environment using a six-dimensional force/torque sensor. As the robot’s end effector approaches the target hole, the sensor detects the force feedback in various directions.

(2) Force/Displacement Model:

Based on admittance control, the robot’s end effector adjusts its motion according to the perceived contact forces. Specifically, when the robot contacts the hole, the control system calculates the desired displacement, which is typically fine-tuned in the vertical direction to ensure precise alignment with the hole.

(3) Vertical Adjustment and Search:

In this process, the robot adjusts its position along the vertical axis (usually the *Z*-axis) and fine-tunes its speed and direction based on real-time feedback, until the end effector is perfectly aligned with the target hole.

(4) Error Reduction:

One significant advantage of the vertical hole search strategy is its ability to reduce the number of search iterations. Through admittance control, the robot’s end effector can quickly and precisely adjust its position based on feedback, thus minimizing the excessive searching that may occur with traditional methods.

Advantages of the Vertical Hole Search Strategy:

Reduced Search Iterations: Compared to traditional search strategies (such as the Archimedean spiral search), the vertical hole search strategy is more efficient in reducing the alignment error and improving search precision. Admittance control enables real-time adjustment of the robot’s posture based on force feedback, allowing the robot to quickly find and mate with the hole.

Improved Precision: By utilizing force feedback and admittance control, the robot can adjust its end effector position according to the contact forces and displacement, enabling high-precision assembly. For the square box assembly, this ensures accurate hole-to-hole alignment.

Real-time Dynamic Adjustment: This strategy allows the robot to dynamically adjust its posture during the assembly process, adapting to different working environments and assembly precision requirements, avoiding the issues caused by fixed paths or error accumulation.

### 3.3. Vertical Hole Search Strategy

**Figure 4:**
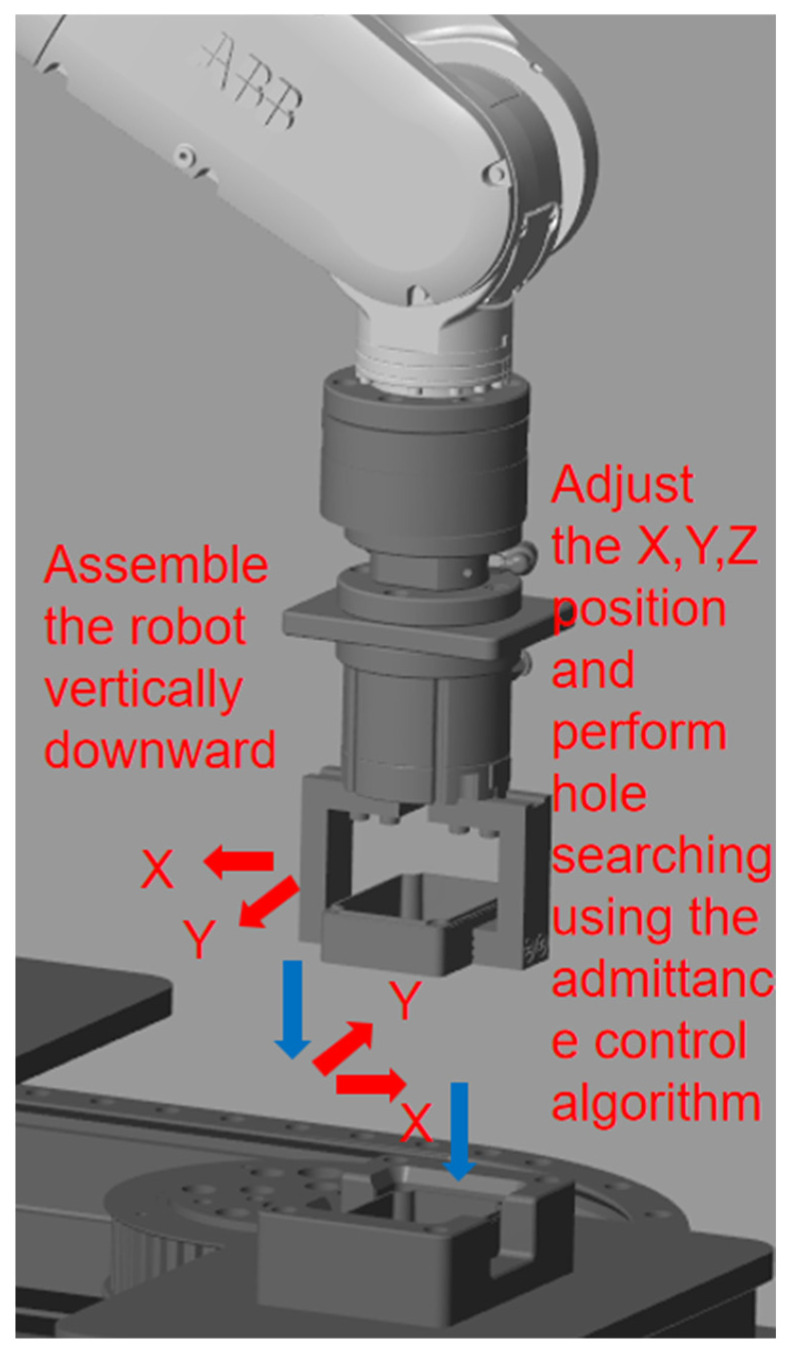
Schematic of the vertical hole searching strategy.

The vertical hole searching strategy involves positioning the robot directly above the target assembly hole using position control, followed by a vertical downward assembly. Displacement corrections are made through the admittance control algorithm, which updates the position to achieve compliance. As shown in Figure 4, this illustrates a schematic of the vertical hole searching process. The vertical hole searching strategy is more suitable for non-typical square holes, irregularly shaped holes, and other complex parts. Its primary advantage is that it eliminates the need to search for contact points, saving significant time in the hole searching process. Additionally, by relying on robot position control, vertical assembly reduces the need for adjustments in multiple directions. In contrast, the Archimedes spiral search adjusts based on contact points, resulting in an initial force greater than that of the vertical hole search, which involves more complex subsequent force feedback corrections for displacement. Therefore, the vertical hole searching strategy can improve work efficiency compared to traditional blind hole searching strategies. From a theoretical standpoint, the Archimedes spiral hole search is inefficient, primarily due to the fact that many invalid trajectory points are encountered during the search process, leading to a decrease in overall assembly efficiency. The vertical hole search strategy proposed in this paper is based on position-controlled trajectory planning. It ensures the gripper moves above the sealed box to the designated assembly position. This strategy avoids multiple passes over invalid points, significantly improving efficiency in terms of time. It is particularly suitable for assembly scenarios involving chamfered components. However, while the contact profile for circular holes is continuous in space and can be analyzed in two dimensions, the contact profile for square holes is discontinuous, necessitating three-dimensional space analysis. In addition to position deviations, there are also angular deviations, which significantly increase the difficulty of the assembly. Therefore, it is necessary to apply admittance control for force feedback to correct displacement deviations during the assembly process.

## 4. Admittance Control and Its Theoretical Foundation

### 4.1. Theoretical Foundation of Admittance Control

This paper employs an admittance-based force control algorithm, where the contact force with the environment is used as input and the position deviation is the output. The robot’s end effector and its interaction with the environment are modeled as an equivalent second-order system composed of a spring–damper–mass model. The displacement compensation is generated in response to external displacement changes to correct for position deviations, ultimately achieving control of the target displacement through the inner-loop displacement. The measured force is transmitted to the robot’s control unit, which adjusts the robot’s pose to achieve compliant control. The mathematical model for admittance control is as follows:

(3) $$M_{d}\ddot{x}+B_{d}\dot{x}+K_{d}x_{e}=F_{e}-F_{d}\;x_{e}=x-x_{d}$$

where $M_{d}$ represents the inertia coefficient matrix, $B_{d}$ represents the damping coefficient matrix, $K_{d}$ represents the stiffness coefficient matrix, $x_{d}$ denotes the desired position, x is the actual position, $F_{e}$ is the actual contact force between the robot and the external environment, and $F_{d}$ is the desired contact force.

Since admittance control is a position-based impedance control strategy, the difference between the actual contact force at the robot’s end effector and the desired force corresponds to the position correction. By transforming the mathematical model, we can derive the following:

(4) xe¨=(F−Bdx˙−Kdx)Md

According to Euler’s method, we can obtain the following:

(5) $${\dot{x_{e}}}^{\left(n+1\right)}={\dot{x_{e}}}^{\left(n\right)}+{\ddot{x_{e}}}^{\left(n\right)}\mathrm{dt}$$

Integrating Equation (5), we obtain the following:

(6) $${x_{e}}^{\left(n+1\right)}={x_{e}}^{\left(n\right)}+{\dot{x_{e}}}^{\left(n+1\right)}\mathrm{dt}$$

In Equations (5) and (6), ${x_{e}}^{\left(n\right)}$ and ${\dot{x_{e}}}^{\left(n\right)}$ are obtained based on the robot’s feedback of the current joint angle θ. By combining Equations (4)–(6), the position correction amount ${x_{e}}^{\left(n+1\right)}$ is obtained. The values in Equation (6) represent the corrected position.

(7) xe(n+1)=xe(n)+xe˙(n)+F−Bdxe˙(n)−Kdxe(n)Mddtdt

**Figure 5:**
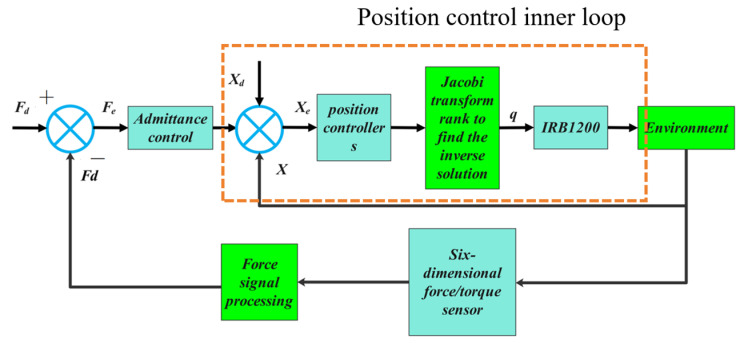
Block diagram of admittance controller.

From Equation (7), it can be seen that the displacement of the robot’s end effector at the next time step is related to the position correction of the current cycle. The block diagram of the admittance control system is shown in Figure 5. In the admittance control block diagram shown above in Figure 5, starting from the right side, when the robot end effector contacts the environment, the force information collected by the force sensor undergoes filtering (currently through noise reduction filtering within the six-dimensional force sensor) and gravity compensation. Afterward, the force is decoupled to obtain the actual contact force at the robot gripper’s end. Then, the force deviation is calculated based on the set expected force. This part is related to the equations introduced in this paper, including the established second-order model, where the contact between the end effector and the environment is modeled as an equivalent mass–damping–spring second-order system.

### 4.2. Admittance Parameter Analysis of Admittance Control

The simulation program’s control block diagram includes the admittance control module, the robot’s forward and inverse kinematics modules, and modules for contact force error and contact force error rate. During the simulation, external environmental interference and communication cycle effects are not considered, and the experiment is conducted in an ideal environment. The simulation experiment will focus on force tracking error and position tracking error, with a comparison of the simulation results.

**Figure 6:**
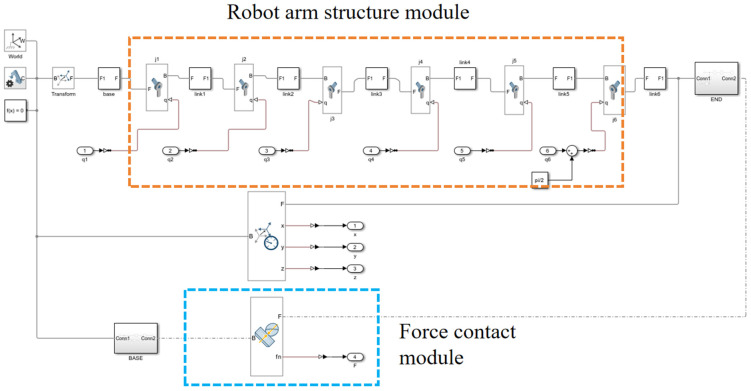
The Simscape model of linkages and joints.

**Figure 7:**
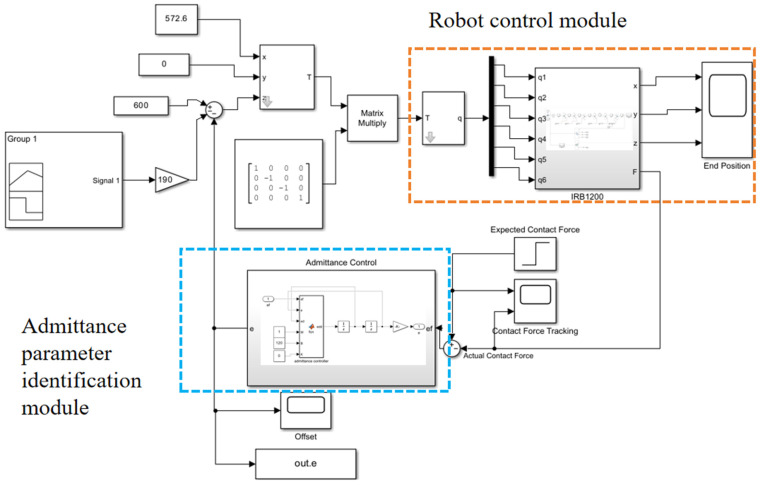
Simulation control system model.

We obtained the robot’s physical parameters through analysis using SOLIDWORKS 2022 and NX 2206. However, due to the closed nature of the robot system, the relevant parameters could not be directly obtained, which results in some discrepancies compared to actual experiments. The Simulink model exports the 3D model of the robotic arm from the 3D software and directly imports the mass and relationship parameters of each joint into Simulink’s dynamics module. The primary source of resistance is the contact force, with friction not being considered. The contact force simulates real-world contact and is modeled through the creation of a contact module, which can be understood as a force sensor. By transforming the coordinate systems between robot joints, the links and joints are connected. The following figure (Figure 6) shows the model for establishing the link and joints:

To accurately control the assembly contact force and position deviation, and to ensure that the contact force is adjusted to the desired range, it is necessary to analyze the admittance parameters. A position-based impedance control simulation model was built in Matlab’s Simulink, as shown in Figure 7. The desired force is set to 20 N, and the analysis of the admittance parameters is conducted using the method of controlling variables.

**Figure 8:**
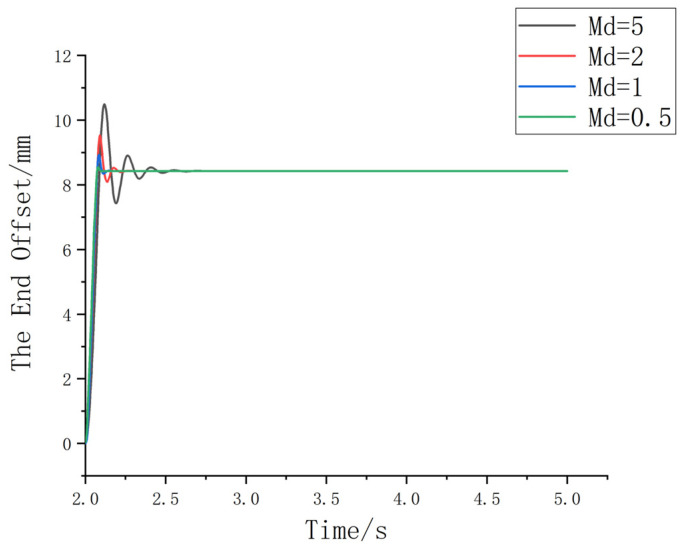
Schematic diagram of the change curve under different inertia parameters.

(1) The damping coefficient $B_{d}$ is set to 120, and the stiffness coefficient $K_{d}$ is set to 0. The value of $M_{d}$ is varied, with settings of 5, 2, 1, and 0.5, as shown in Figure 8.

Based on Figure 8, since the inertial parameters are related to the acceleration of the robot’s end effector, a large value may result in slower response time, increased overshoot, and other undesirable effects. Considering the actual assembly conditions, in order to achieve a system with minimal overshoot, short response time, small fluctuations, and stable performance, a reasonable $M_{d}$ value should be selected. In this case, after comprehensive consideration, it is chosen to be 1.

**Figure 9:**
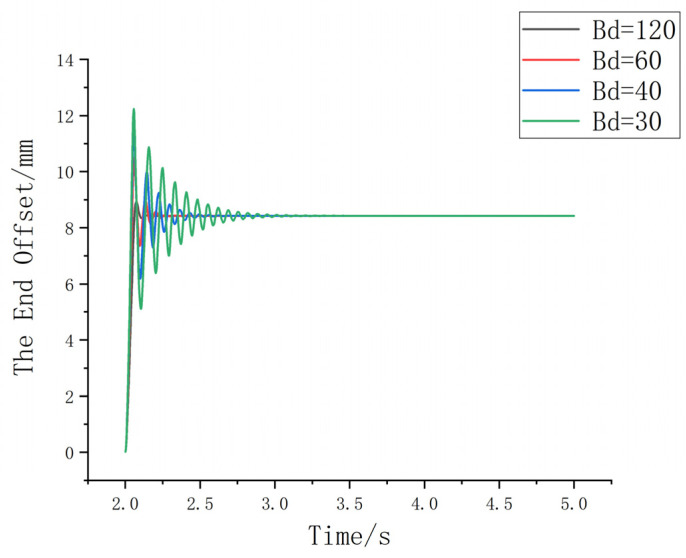
Schematic diagram of the change curve under different damping parameters.

(2) With $M_{d}$ set to 1 and $K_{d}$ set to 0, the value of $B_{d}$ is adjusted to 120, 60, 40, and 30, as shown in Figure 9.

Based on Figure 9, since the damping parameter is related to the energy of the system, a large value may lead to slower response time, increased overshoot, and other undesirable effects. Considering the actual assembly conditions, in order to achieve a system with minimal overshoot, short response time, small fluctuations, and stable performance, a reasonable $M_{d}$ value should be selected. After comprehensive consideration, $B_{d}$ is chosen to be 120.

**Figure 10:**
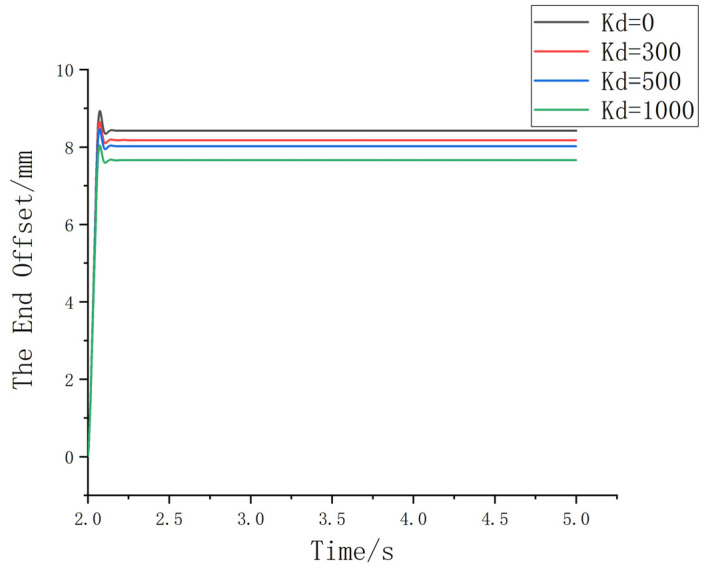
Schematic diagram of the change curve under different stiffness parameters.

(3) With $M_{d}$ set to 1 and $B_{d}$ set to 120, the value of $K_{d}$ is adjusted to 0, 300, 500, and 1000, as shown in Figure 10.

**Figure 11:**
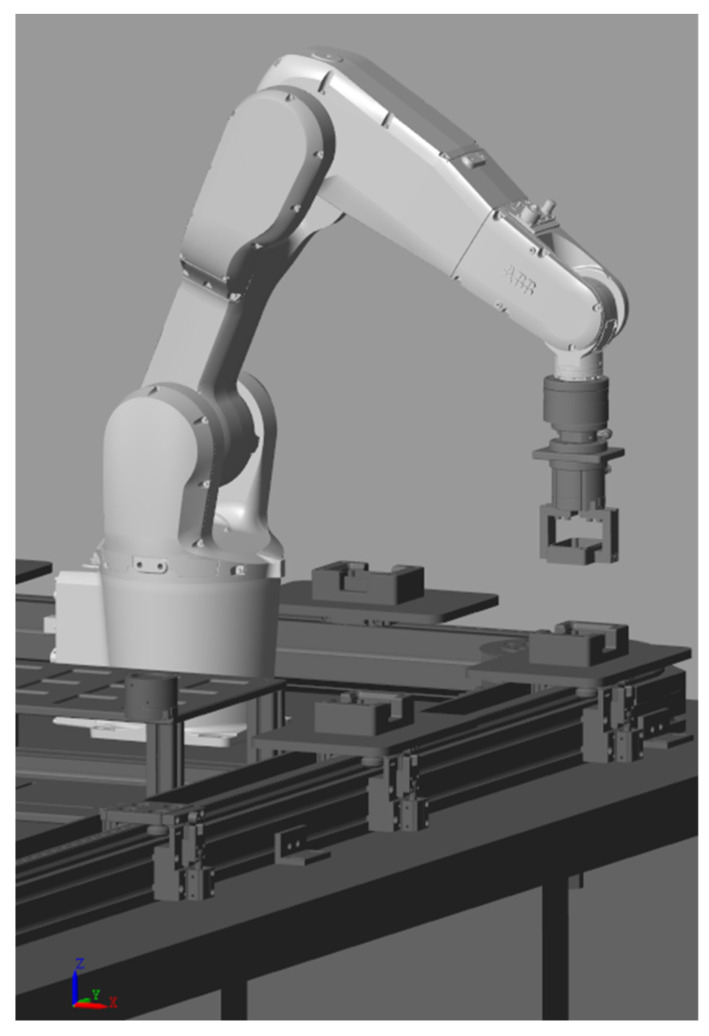
Simulation of hole shaft assembly under admittance control.

Based on Figure 10, since the damping parameter affects the end effector’s pose correction, the stiffness parameter settings vary depending on the specific scenario. For situations with high sensitivity, lower stiffness parameters are more suitable, while for other scenarios, higher stiffness parameters are preferred. In the context of this assembly setup, a lower $K_{d}$ value should be chosen. After comprehensive consideration, $K_{d}$ is set to 0. By selecting optimal admittance parameters, the contact force and positional deviation between the shaft and hole can be precisely controlled, ensuring that the contact force is adjusted within an appropriate range. The final settings are ($M_{d}$ = 1), ($B_{d}$ = 120), and ($K_{d}$ = 0). To verify this, the assembly process is simulated using the Simulink module. Figure 11 shows the simulation of the shaft–hole assembly under admittance control.

**Figure 12:**
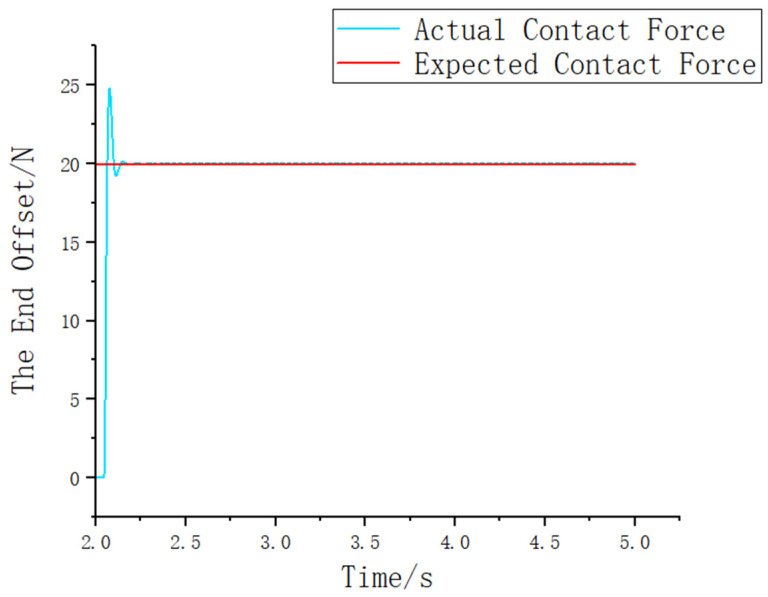
Assembly force tracking curve.

As shown in Figure 12, at the 2 s mark, the robot end effector makes contact with the constrained environment. Using the compliance control algorithm, the assembly force is reduced from the maximum contact force of 24.76 N to 20.01 N at 2.25 s, with minimal impact during the process. The overall force tracking performance is clearly evident, and the compliance control effect is significant, aligning with the expected experimental results.

Through simulation experiments, we obtained the appropriate Admittance parameters using the control variable method and tested their performance through force tracking simulations. The results generally meet the requirements for the assembly of the square box and square groove stations. Smaller Admittance parameters indicate the stability of the second-order system. Therefore, the simulation experiments are essential and advantageous for the subsequent development of compliant assembly.

## 5. Square Hole Compliance Assembly Experiment

### 5.1. Hardware of the Control System

**Figure 13:**
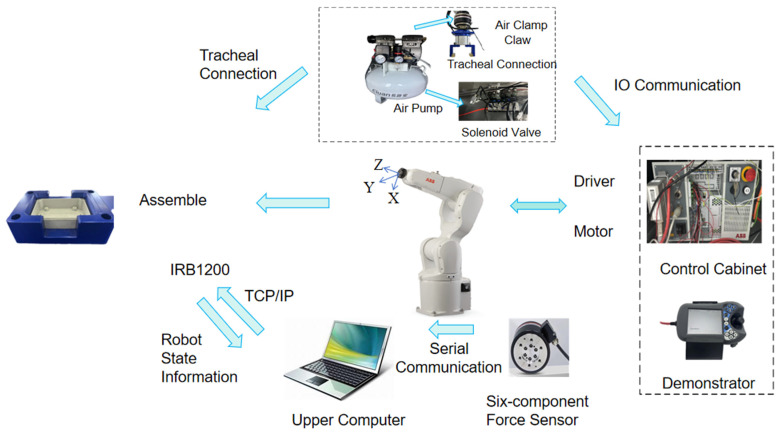
Control system hardware part.

Figure 13 illustrates the hardware components of the control system used in this study. To complete the robot assembly of a square seal box to a square groove station, a six-dimensional force/torque sensor is integrated between the robot’s end effector and the pneumatic gripper. This sensor is used to detect the contact force and torque during the assembly process, and is securely mounted via a custom flange with threaded fastening. The pneumatic gripper is powered by an air pump, with functionality controlled via an electromagnetic valve and I/O signals. The data collected by the six-dimensional force sensor are transmitted to the host computer via serial communication. Communication between the robot and the host computer is established through TCP/IP. The pneumatic cylinder of the gripper is an AIRTAC HFC150, the gripper itself is a custom design, and the six-dimensional force sensor is the Desente γ74 series six-axis force sensor. The square groove station is also designed in-house.

Table 1 and Table 2 show the performance parameters and motion parameters of the IRB1200 robot, respectively.

**Figure 14:**
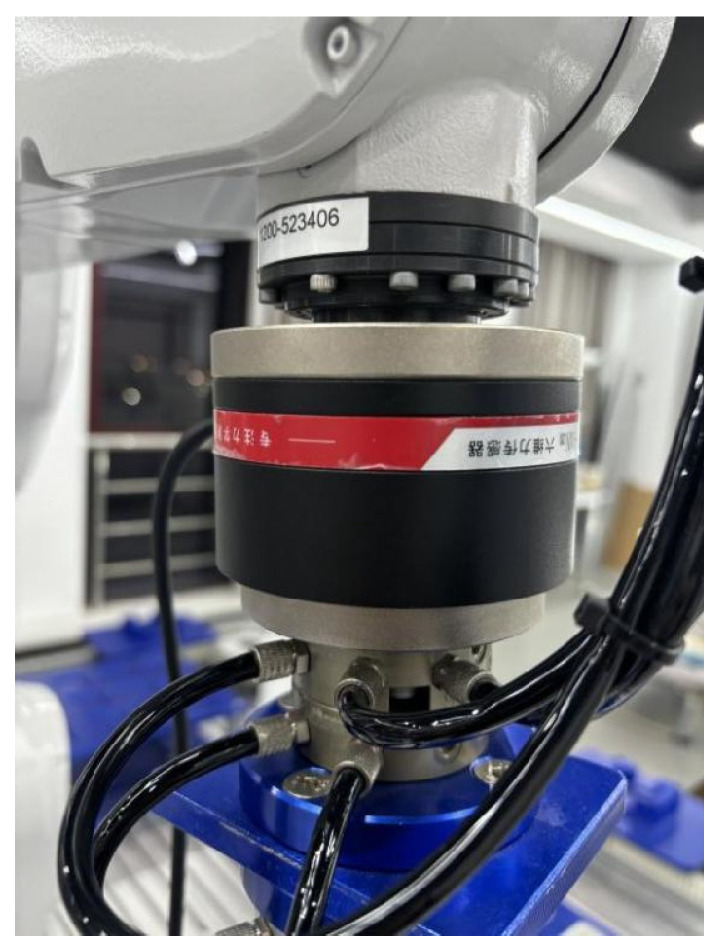
Six-dimensional/torque sensor actual installation position.

The installation of the force sensor is shown in Figure 14 below. For industrial robots, in order to collect contact force information through the six-axis/torque sensor, the sensor is typically placed between the end effector and the robot’s sixth axis. Table 3 shows the mechanical characteristics of the six-axis force sensor.

### 5.2. Sensor Gravity Compensation Experiment

**Figure 15:**
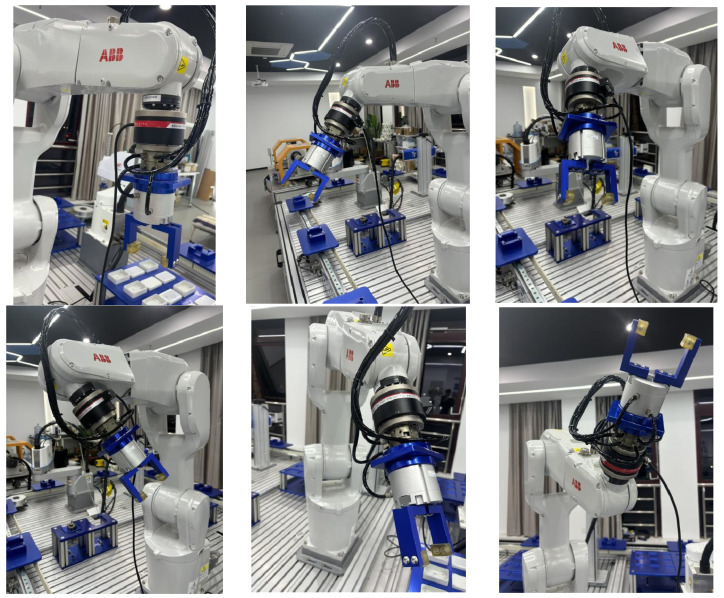
IRB1200 random pose gravity compensation plot.

When the robot performs precision tasks, such as assembly, accurately perceiving the six-dimensional force and torque at the end effector’s contact with the external environment is crucial. This force feedback allows the robot to adjust its posture in real time, which is key to achieving force-sensitive manipulation. At the robot’s end effector, the six-dimensional force sensor experiences a bias in its output due to the weight of the end tool and the sealing box. This inevitably requires compensating for gravitational force. Therefore, to verify the necessity and feasibility of the compensation, the robot is moved into random postures to simulate gravitational variations during its movement. The experiment collects force signals at different states with the six-dimensional force/torque sensor. Figure 15 below shows the data collection experiment at various positions.

**Figure 16:**
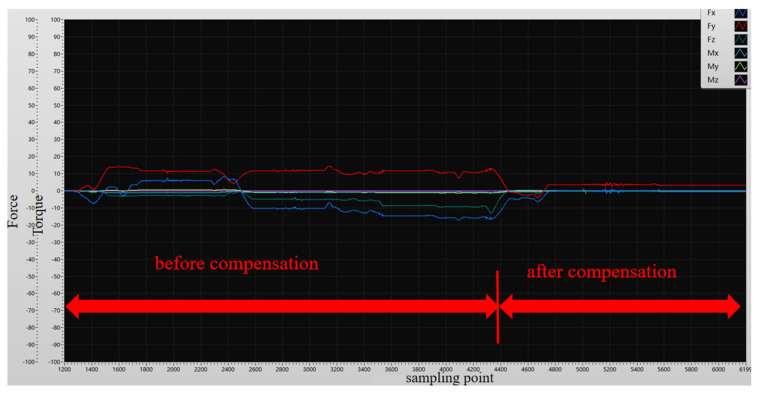
Sensor compensation process diagram.

In different postures, the robot undergoes data compensation with the six-dimensional force/torque sensor. As shown in Figure 16, the forces and torques in the *X*, *Y*, and *Z* axes stabilize near 0, indicating that the gravity compensation algorithm proposed earlier is feasible.

**Figure 17:**
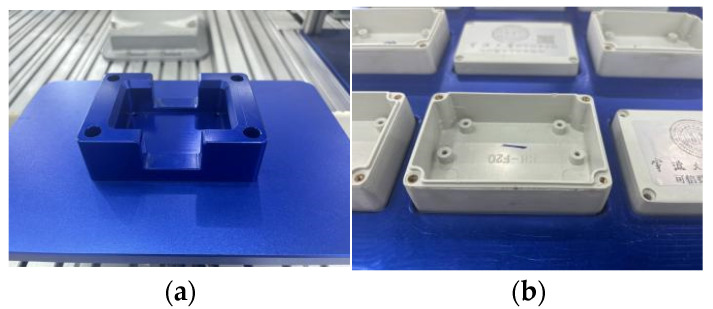
The square groove station and the sealed box to be assembled. (**a**) Square slot station. (**b**) Sealed box.

In the experiment on the ring-shaped production line shaft–hole compliant assembly task, this paper incorporates the actual assembly of the square sealing box and the square groove station in the laboratory, as shown in Figure 17a,b. In the experiment, considering the interference of external factors, it is assumed that the gripper captures the shaft in the same position each time. This ensures that the torque error when the shaft and hole contact is minimal. At the same time, it is necessary to ensure that the vertical hole searching range and the pitch of the spiral hole searching are within the clearance fit range between the shaft and the hole. The shaft–hole fit relationship is a clearance fit. The dimensions of the sealed box to be assembled are 82.3 mm in length and 57.3 mm in width, while the square groove station measures 82.4 mm in length and 57.4 mm in width. The fitting clearance between the components is ±0.1 mm, as shown in Figure 17.

### 5.3. Robot Communication Section

Due to the closed nature of the ABB control box, the basic approach is to first transmit the force information obtained from the six-axis force sensor and the robot’s state information (such as pose, velocity, etc.) to the PC. All gravity compensation and force control algorithms are executed on the PC, and the computation results (robot motion poses) are then transmitted to the robot via communication, which drive its motion. The entire upper-level program is written in Matlab. To complete the compliant assembly task, the upper-level computer must continuously communicate with the ABB IRB1200 to transfer information and control motion. The PC is connected to the ABB IRB1200 via Ethernet for information transfer. Communication is implemented using the TCP/IP socket method, where the client and server are the two communicating parties. Robot status information is retrieved in real time via TCP/IP. The force sensor is connected to the PC via USB, and force data during the assembly process are collected in real time through TCP/IP.

The communication details between the IRB1200 robot and the PC can be described in two aspects: one being the communication delay and the other being an example code snippet for real-time control. The goal is to demonstrate how to implement real-time force feedback and pose adjustments for the robot. Communication Delay: Tests show that the average delay for TCP/IP communication is 20 milliseconds, which is compensated for by a real-time PID control algorithm, ensuring that the response time remains within 50 ms.

**Figure 18:**
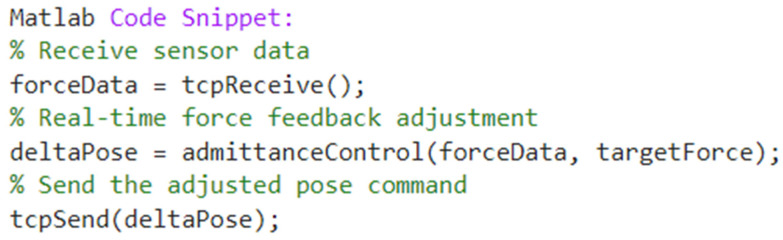
Matlab code snippet.

The following Figure 18 is an example code snippet for real-time control.

### 5.4. Compliant Assembly Process

**Figure 19:**
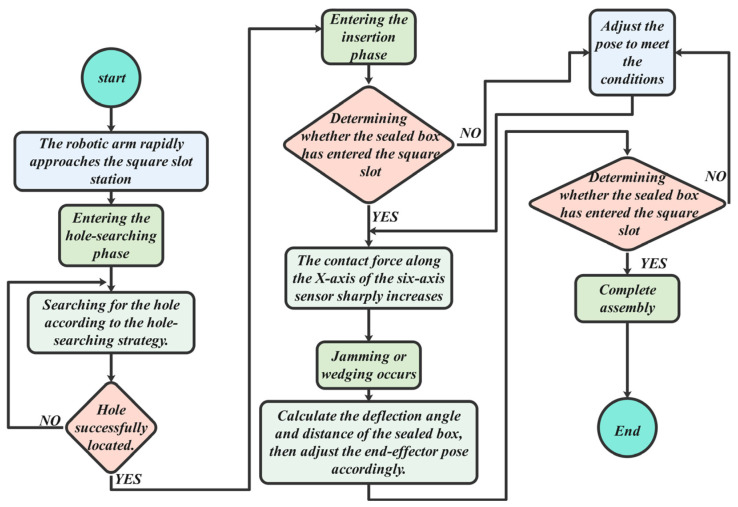
Compliant assembly flow chart.

The square hole compliant assembly process is shown in Figure 19, with the core stages being the hole searching phase and the insertion adjustment phase. Initially, the robot quickly moves to the vicinity of the square groove station following trajectory planning. It then enters the hole searching and contact adjustment phase, where the Archimedes spiral search strategy and vertical hole searching strategy are compared. When the *Z*-axis force reaches the preset threshold or the *Z*-axis position moves 1 mm in the negative direction, the new position is updated. At this stage, the robot uses an admittance control algorithm to perform shaft–hole compliant assembly, overcoming issues like jamming or wedging.

**Figure 20:**
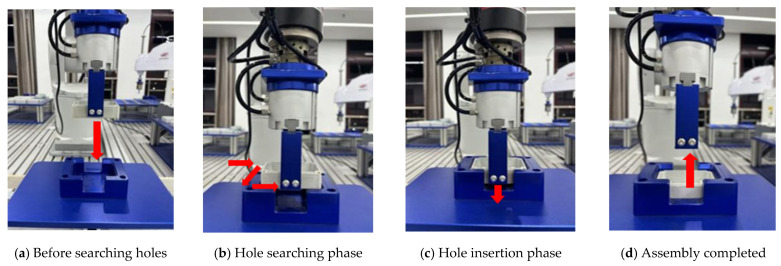
The compliant assembly process of the hole shaft under the vertical hole searching strategy for Case A.

Once the square sealing box is correctly aligned with the square groove station, the process advances to the insertion phase. If alignment cannot be achieved, the robot retracts to the original position for re-adjustment. After successful insertion, the robot releases the pneumatic gripper, completing the compliant assembly process. The end of the hole searching phase marks the completion of the compliant assembly. Figure 20 below illustrates the hole–axis compliant assembly process of hole–axis compliant assembly in Case A. Case B, serving as the control group, conducts the experiment based on the Archimedean spiral hole searching method described in Section Typical Failure Analysis of Square Hole Assembly. Hole searching is carried out along the predefined spiral trajectory, while ensuring that the pitch of the spiral hole searching remains within the clearance fit range between the shaft and the hole.

The assembly process consists of four stages. This paper focuses on investigating the impact of different impedance parameters during the insertion of square holes on the compliance of hole searching, as well as the hole searching efficiency under different searching strategies. At the beginning of the hole searching process, an initial force of 1 N is set in each direction using admittance control.

**Figure 21:**
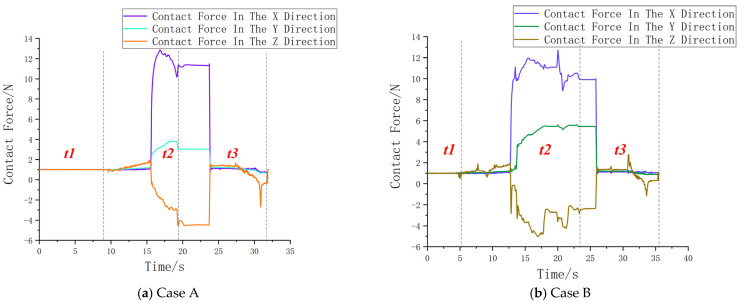
The variation chart of the three-dimensional component contact force for Case A and Case B.

Figure 21a shows the variation in the three-dimensional contact force during the compliant assembly process under the vertical hole searching strategy. The entire experiment is divided into three stages: the first stage is the approach phase, denoted as t1 in the diagram; the second stage is the hole searching phase, denoted as t2 in the diagram; and the third stage is the hole insertion phase, denoted as t3 in the diagram. At the 16th second of the assembly experiment, the robot begins to apply significant force contact during the hole searching task. Around the 16 s mark of the assembly experiment, the robot completes the hole searching task and begins the insertion phase. From the image, it is clear that a significant jamming occurs in the X direction, but the jamming force quickly drops from 13 N to 11 N, after which the insertion continues. By the 24th second of the experiment, the hole searching task is completed, and the hole searching phase is 10 s, and the robot then begins lowering the sealing box before returning to the origin.

Figure 21b shows the compliant control assembly under the spiral hole searching strategy. Clearly, compared to the vertical strategy, this approach exhibits lower hole searching efficiency, with frequent jamming and slower response. As observed in the continuous peaks in the figure, the contact force reaches 13 N at the 20th second, decreases sharply to 9 N at the 21st second, and then gradually increases before stabilizing, with a maximum drop of 4 N during this period.

**Figure 22:**
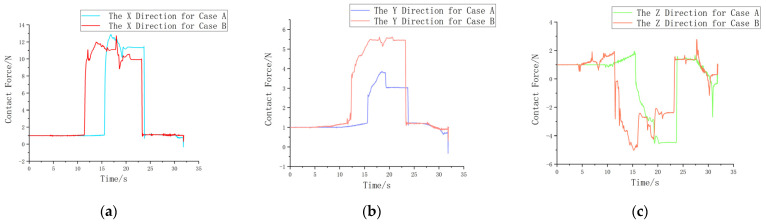
The comparison chart of the variation in contact force for each component in Case A and Case B. (**a**) The F_x_ contact force in Cases A and B. (**b**) The F_y_ contact force in Cases A and B. (**c**) The F_z_ contact force in Cases A and B.

Based on the analysis of the contact forces in the components shown in Figure 22, a comparison of the contact forces in the X, Y, and Z directions for each group of the two hole searching strategies shows that, during the hole searching adjustment phase, the improved vertical search strategy takes less time than the Archimedes spiral search strategy. The robot can quickly adjust the hole searching position, making the vertical search strategy perform better in our application compared to the Archimedes spiral search strategy. Additionally, it has faster response times and fewer occurrences of jamming.

**Figure 23:**
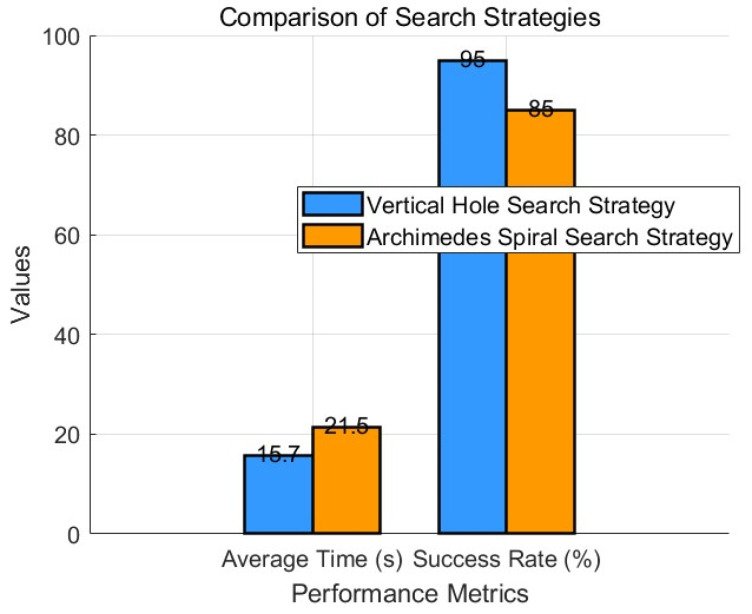
Comparison of vertical hole search strategy and Archimedes spiral search strategy.

To further verify the scientific validity and rigor of the experimental results, the number of trials was raised to 10. The trial counts were recorded, and statistical analysis was conducted. A *t*-test was then used to compare the experimental performance of the vertical hole searching strategy and the Archimedean spiral hole searching strategy. Table 4 below shows a comparison of the experimental data. The following bar chart (Figure 23) clearly illustrates the differences between the two strategies across various performance metrics. The improved vertical hole search strategy outperforms the Archimedes spiral strategy in both average time and success rate.

The *t*-test results confirmed that the improved vertical hole searching strategy significantly outperforms the Archimedean spiral hole searching strategy in terms of search adjustment time (*p* < 0.05). Therefore, the advantages of the improved vertical search strategy are evident. Compared to the traditional Archimedes spiral hole search strategy, the average search adjustment time was reduced by 5.8 s, improving efficiency by 46.4%.

## 6. Discussion

This paper first analyzes the current state of research and unresolved issues in the industry regarding the assembly of square slots and square parts. Based on this, the study is conducted, starting with the simulation of hole–axis assembly using MATLAB Simulink, with a focus on admittance parameter identification. The optimal admittance parameters are obtained through the control variable method, though the appropriate parameters must be selected based on specific operating conditions. Since there are certain differences between the simulated robot model and the actual robot, we need to compare the simulation results with the experimental results. By using a specific comparison table, we can highlight the similarities or differences between the simulated and experimental outcomes. The comparison table is shown in Table 5 below.

From the table, it can be seen that the adjustment rules for the admittance parameters used in the simulation can be directly applied to the experiment, providing guidance for the experimental setup. However, the parameters set in the simulation should not be directly applied to the experiment, as the robot model in the simulation differs from the actual robot in terms of parameters such as mass. There are also some differences in the adjustment range of the contact force between the simulation settings and the actual experiment. Nevertheless, the trends remain consistent. In the experiment, mechanical friction contributes to this discrepancy, as obstructions can cause a significant increase in the contact force. Additionally, the nonlinear behavior of the simulation system (such as friction) has not been fully modeled, which results in differences between the simulation and actual experimental results.

Previous research often used hole searching strategies such as the Archimedes spiral search or random search. However, considering that this study involves square hole assembly, which is a non-typical scenario, a more efficient direct hole searching strategy was adopted in subsequent experiments, offering higher efficiency.

The specific advantage of the vertical hole searching strategy lies in its ability to save hole searching time, as it eliminates the need for spiral blind searching. However, this approach requires precise positioning, such as the use of intelligent algorithms for trajectory planning to reach the target point during the hole searching phase. The strengths and limitations of vertical hole searching are thus discussed. While it offers advantages, it also has certain limitations in different scenarios, and the vertical hole searching strategy should be selected according to the specific application conditions.

The improved vertical hole searching strategy proposed in this paper significantly enhances the accuracy of hole assembly by optimizing the robot’s positioning precision. In particular, it addresses the positioning errors caused by the complexity of the environment in circular production lines, which are a limitation of traditional methods. By incorporating a real-time feedback mechanism into the admittance control algorithm, the robot can flexibly adjust its force and position to adapt to varying assembly conditions and workpiece tolerances. Experimental results showed that this method reduces the error rate to less than 10% of that in traditional methods for high-precision assembly tasks, effectively improving both assembly accuracy and production efficiency on the production line.

## 7. Conclusions

To address the issue of hole–axis assembly in production lines, this paper focuses on the hole searching strategy during the search phase and the identification of admittance parameters during the insertion of square holes. This paper compares various hole searching strategies and analyzes the impact of the admittance parameters $M_{d}$, $B_{d}$, and $K_{d}$ on compliance control assembly through the controlled variable method. The following conclusions are drawn:

(1) Through system analysis, the effects of the three parameters—$M_{d}$, $B_{d}$, and $K_{d}$—on the system’s characteristics were analyzed, and the adjustment rules were summarized. These rules were then applied in the experiment. Admittance parameters affect the system’s stability to varying degrees. Based on the actual assembly process requirements and through the comparison of the three simulations, it was found that, when other parameters remain unchanged, the system’s step response speed slows down as $M_{d}$ increases, while the steady-state error remains largely unchanged. The damping parameter $B_{d}$ influences the system’s stability; as $B_{d}$ decreases, the system becomes more stable. The stiffness parameter $K_{d}$ is dependent on the sensitivity required for external force control, with a smaller $K_{d}$ value being preferred for more stringent force requirements.
(2) To demonstrate the advantages of the improved vertical hole searching strategy proposed in this paper, 10 experimental trials were conducted to test the average hole search contact adjustment time and calculate the success rate. A detailed statistical analysis was then performed, and a t-test confirmed that the improved vertical hole searching strategy significantly outperformed the Archimedean spiral hole searching strategy in both hole search adjustment time and success rate (*p* < 0.05). Therefore, the advantages of the improved vertical search strategy are confirmed. Compared to the traditional Archimedes spiral hole search strategy, the average search adjustment time was increased by 5.8 s, improving efficiency by 46.4%.

By comparing the simulation and experimental results, it is evident that the direct hole searching strategy offers significant advantages in the non-typical square hole assembly scenario. Moreover, selecting the most appropriate admittance parameters has a substantial impact on compliance control assembly, improving assembly speed, efficiency, and success rate. It is also clearly observed that the Archimedes spiral hole search strategy is more prone to jamming compared to vertical search. Additionally, under admittance control, the system can adjust the contact force, offering certain advantages over vision-guided assembly operations.

## Figures and Tables

**Table 1:** Table of IRB1200 robot performance parameter.

1 kg Pick-and-Place Cycle Performance Parameters	
Maximum TCP Acceleration	35 m/s·s
Acceleration Time from 0 to 1 m/s	0.06 s
Repetition Accuracy	0.025 mm

**Table 2:** Table of IRB1200 robot motion parameter.

Axis Movement	Working Range	Maximum Axis Speed
Axis 1 (Rotation)	+170° to −170°	288 °/s
Axis 2 (Arm)	+135° to −100°	240 °/s
Axis 3 (Arm)	+70° to −200°	300 °/s
Axis 4 (Wrist)	+270° to −270°	400 °/s
Axis 5 (Bend)	+130° to −130°	405 °/s
Axis 6 (Twist)	+400° to −400°	600 °/s

**Table 3:** Table of six-dimensional force/torque sensor mechanical characteristics.

Mechanical Characteristics	
Weight	800 g
Dimensions	Φ125 mm × 67.5 mm
Overload Capacity	300% FS
Zero Drift	0.2% FS/10 °C
Sensitivity Drift	0.1% FS/30 min

**Table 4:** Experimental data comparison table.

Search Strategy	Average Search Time (s)	Success Rate (%)	Standard Deviation	Confidence Interval (95%)	*p*-Value
The improved vertical search strategy	15.7	95	0.5	[15.2, 16.2]	*p* < 0.05
Archimedes spiral search strategy	21.5	85	1.2	[20.3, 22.7]	

**Table 5:** Comparison and analysis of differences between simulation results and actual results.

Parameter	Simulation Result	Experiment Result	Difference Analysis
Contact Force Adjustment Range (N)	4.75	6.62	The contact force increases significantly in the experiment due to slight mechanical friction and blockage, leading to a higher contact force.
Impedance Parameter Adjustment Rules ($M_{d}$ $B_{d}$, $K_{d}$)	Consistent trends	Consistent trends	No difference in the adjustment pattern, but since the actual robot is not exactly the same as the simulation, experimental parameters are set based on the simulation’s pattern.

## Data Availability

The datasets used and/or analyzed during the current study are available from the corresponding author on reasonable request.
